# Study on expression of lncRNA RGMB-AS1 and repulsive guidance molecule b in non-small cell lung cancer

**DOI:** 10.1186/s13000-015-0297-x

**Published:** 2015-06-09

**Authors:** Ping Li, Juan Li, Rui Yang, Furui Zhang, Huaqi Wang, Heying Chu, Yao Lu, Shaozhi Dun, Yuanyuan Wang, Wenqiao Zang, Yuwen Du, Xiaonan Chen, Guoqiang Zhao, Guojun Zhang

**Affiliations:** Department of Respiratory Medicine, The First Affiliated Hospital of Zhengzhou University, Zhengzhou, 450052 China; Department of Microbiology and Immunology, College of Basic Medical Sciences, Zhengzhou University, Zhengzhou, 450001 China; Emergency Department, Zhengzhou Central Hospital Affiliated to Zhengzhou University, Zhengzhou, 450007 China

**Keywords:** Non-small-cell lung cancers (NSCLC), lncRNA RGMB-AS1, Repulsive guidance molecule b (RGMB)

## Abstract

**Background:**

The relationships between lncRNAs and tumors have currently become one of the focuses on cancer studies. However, there are a few studies about lncRNAs in non-small cell lung cancer (NSCLC) at present.

**Methods:**

Microarray analysis was designed to study the expression patterns of lncRNAs in three pairs of NSCLC tissues. The expression of lncRNA RGMB-AS1 and repulsive guidance molecule b (RGMB) were detected in 72 paired NSCLC tissues and adjacent normal tissues by qRT-PCR assay. The relations of lncRNA RGMB-AS1 and RGMB expression with clinicopathological factors of NSCLC patients were explored. A549 and SPC-A-1 cells were transfected with siRNA of lncRNA RGMB-AS1 and negative control. RGMB expression level was detected by qRT-PCR assay and western blot analysis.

**Results:**

The results of microarray found that 571 lncRNAs were differentially expressed in NSCLC tissues (Fold change cut-off: 5.0, *P* < 0.05), including 304 upregulated and 267 downregulated lncRNAs. The results of qRT-PCR showed that lncRNA RGMB-AS1 expression was significantly higher in NSCLC tissues than in adjacent normal tissues (*P* < 0.05), while RGMB mRNA showed an opposite trend (*P* < 0.05). Correlation analysis indicated that the expression of lncRNA RGMB-AS1and RGMB mRNA were inversely correlated (R^2^ = 0.590, *P* < 0.05). While lncRNA RGMB-AS1 and RGMB expression levels in NSCLC tissues were associated with the occurrence of differentiation status, lymph node metastases and TNM stage (*P* < 0.05). Transfection with siRNA of lncRNA RGMB-AS1, subsequent results showed that RGMB mRNA and protein expression were upregulated (*P* < 0.05) in A549 and SPC-A-1 cells compared to the control groups.

**Conclusion:**

We identified lncRNA RGMB-AS1 was upregulated and RGMB was downregulated in NSCLC patients. Both were related to differentiation status, lymph node metastases and TNM stage. Studies also indicated that lncRNA RGMB-AS1and RGMB were inversely correlated.

**Virtual slides:**

The virtual slide(s) for this article can be found here: http://www.diagnosticpathology.diagnomx.eu/vs/7911587521528276

## Background

Lung cancer, one of the most frequent malignancies in the world, is rapidly becoming the main cause of cancer related death nowadays [1]. Approximately 85 % of patients are diagnosed with non-small cell lung cancer (NSCLC). Modifications of chemotherapy combinations, the addition of monoclonal antibodies, including bevacizumab [2, 3] and cetuximab [4, 5], and the incorporation of histologic subtype [6] into treatment decisions have added to the survival of patients with advanced NSCLC. Unfortunately, therapeutic outcomes appear to have reached a plateau, with response rates of 20 to 35 % and median survival of 8 to 12 months [7]. Currently, encouraging new targeted agents have led to a better understanding of the molecular pathogenesis that causes NSCLC, especially the non-protein-coding portion (~90 %) as non-coding RNA (ncRNA) of the genome [8]. The ncRNAs characterize as three types, long ncRNAs, mid-size ncRNAs and short ncRNAs [9]. Although most studies on ncRNAs are focused on short ncRNAs, such as microRNAs (miRNAs) [10], long non-coding RNAs (lncRNAs) are rapidly gaining prominence recently.

LncRNAs, tentatively defined as non-coding RNAs more than 200 nucleotides (nt) [11], are usually divided into exonic lncRNAs, intronic lncRNAs, intergenic lncRNAs and overlapping lncRNAs in accordance with their location relative to the protein-coding transcripts [12]. In recent years, a large number of lncRNAs have been identified and the abnormal expression of lncRNAs has been implicated in imprinting [13], enhancing various biological functions [14], X chromosome inactivation, charomatin structure [15]. Thus, lncRNAs are critical for normal development and, in many cases, are deregulated in diseases, such as cancer [16–19].

Currently, studies on lncRNAs in NSCLC are limited. In this study, we analyzed the expression patterns of lncRNAs in three pairs of NSCLC tissues. With performing qRT-PCR assay, we chose some kinds of lncRNAs to test and verify the results of microarray analysis. Combined with the UCSC data-base and bioinformatics analysis, not all lncRNAs from microarray showed potential significance. With the screening tests, we found lncRNA RGMB-AS1 may have potential function in the development of NSCLC. So we focused on the expression of lncRNA RGMB-AS1, and analyzed expression of its potential protein on the base of their location. Then the relationships between the expression of lncRNA RGMB-AS1 and repulsive guidance molecule b (RGMB) and clinicopathological factors of patients with NSCLC were explored. Our results may provide a new perspective for the mechanisms of NSCLC.

## Methods

### Patients and tissue samples

This study was approved by the Ethics Committee of Zhengzhou University. Seventy-two paired NSCLC tissues and adjacent normal tissues (≥3 cm away from tumor) were obtained from patients who received surgical resection of NSCLC between 2012 and 2014 in the First Affiliated Hospital of Zhengzhou University. Of the seventy-two pairs of samples, three were used in lncRNA microarray analysis and all pairs were used for additional evaluations. Patients were diagnosed with NSCLC was confirmed by histopathology. The tumor samples and matched adjacent normal tissues were snap-frozen in liquid nitrogen immediately after resection until total RNA extraction.

### Cell culture and transfection

Human NSCLC cell lines (A549 and SPC-A-1) were obtained from the Type Culture Collection of the Chinese Academy of Sciences (Shanghai, China). Both were cultured in RPMI-1640 medium (Gibco BRL, Grand Island, NY) supplemented with 10 % fetal bovine serum, 100 U/mL penicillin, and 100 μg/mL streptomycin at 37 °C in a humidified 5 % CO_2_ atmosphere. In all experiments, exponentially growing cells were used.

For transfection, cells were seeded into six-well plates at a density of 5 × 10^4^ cells/well. When cells viability reached approximately 80 %, transient transfection was performed using Lipofectamine™ 2000 (Invitrogen, Carlsbad, CA, USA) following the manufacturer’s instructions. siRNA, working on lncRNA RGMB-AS1, and control oligonucleotide (negative control) were synthesized by Shanghai GenePharma Co. Ltd.

### RNA extraction

For NSCLC tissues, if the proportion of NSCLC cells in a tissue section was >80 % then the frozen block was subjected to RNA extraction. According to the manufacturer's protocol, total RNA was extracted from 72 pairs of snap-frozen NSCLC tissues and adjacent normal tissues using TRIzol reagent (Invitrogen, CA, USA). For NSCLC cell lines, total RNA was extracted with an RNA Extraction Kit (Qiagen). The integrity of the RNA was evaluated by a Nanodrop ND-1000 (Thermo Scientific, Worcester, USA). The value of OD260/280 is around 1.8 as a criterion of acceptable purity.

### Microarray analysis

Of the seventy-two pairs of samples, three pairs of matched samples were used for microarray analysis. RNA was purified from total RNA after removal of rRNA (mRNA-ONLY™ Eukaryotic mRNA Isolation Kit, Epicentre). Then, each sample was transcribed into fluorescent cRNA along the entire length of the transcripts without 3’ bias utilizing a random priming method. The labeled cRNAs were hybridized onto the Human lncRNA Array v2.0 (8 × 60 K, Arraystar).

### qRT-PCR analysis

For qRT-PCR assay, RNA was reverse transcribed to cDNA from 1.0 μg of total RNA using a Reverse Transcription Kit (Thermo Scientific),. Real-time PCR analyses were conducted using the Power SYBR Green (Thermo Scientific). All protocols were carried out according to the manufacturer's instructions. LncRNA RGMB-AS1 (NR_033932) and RGMB mRNA (NM_001012761) expression were determined by qRT-PCR. GAPDH mRNA (NM_002046) served as an endogenous control for normalization. The correlating primers are the following sequences: lncRNA RGMB-AS1, forward: 5’AGTGGGCAAACTTCAACGTTC 3’, and reverse: 5’ GAGCTGCCATTGAATTAATCCG 3’. RGMB, forward: 5’TTCAGGTTCAAGTGA CAAACG-3’ and reverse: 5’-ACTGAACCTGACCGTACATCATCTGTCACAGCT TGGTA -3’. GAPDH, forward: 5’-AGAGGCAGGGATGATGTTCTG-3’, and reverse: 5’-GACTCATGACCACAGTCCATGC-3’. The PCR reaction was executed on an ABI 7500 Fast Real-Time PCR System (Applied Biosystems, CA, USA) at 95 °C for 180 s, followed by 40 cycles of 95 °C for 15 s and 65 °C for 30 s. All experiments were performed in triplicate, and melting curve analysis was performed to ensure the specificity of the products after amplification. The median in each triplicate was used to calculate relative LncRNA RGMB-AS1 and RGMB mRNA concentrations (ΔCt = Ct median lncRNA or mRNA - Ct median GAPDH), which was then converted to x-fold changes(2^-ΔCt^) [20].

### Western blot analysis

Total proteins were extracted from the transfected cells at 48 h after transfection and then were subjected to SDS-PAGE (SDS/polyacrylamide (10 %) gel electrophoresis) and transferred electrophoretically onto polyvinylidene difluoride (PVDF) filter membranes (Whatman). The membranes were blocked in 5 % skim milk for 1 h, washed four times with Tris-buffered saline containing Tween 20 (TBST) at room temperature, then incubated overnight at 4 °C with diluted primary antibody (rabbit anti-human RGMB antibody, 1:500, Santa Cruz Biotechnology). Following extensive washing with TBST, the membranes were incubated with secondary antibody (goat anti-rabbit IgG, 1:2000, Santa Cruz Biotechnology) for 1 h. After four washes (15 min each) with TBST at room temperature, the immunoreactivity was visualized by enhanced chemiluminescence. The antibody against GAPDH (Santa Cruz Biotechnology) served as an endogenous reference.

### Statistical analysis

SPSS17.0 soft-ware (SPSS, Inc, Chicago, IL) was performed for statistical analysis. Data were expressed as the mean ± the standard deviation (SD). The student’s t-test and a one-way analysis of variance (ANOVA) were used in the comparison of means from different samples. *P* values of less than 0.05 were considered statistically significant.

## Results

### Overview of different lncRNA expression profilings in NSCLC tissues relative to adjacent normal tissues

We profiled lncRNAs expression in tumors from NSCLC patients. Comparing to adjacent normal tissues on the basis of lncRNA array results, we found that 571 lncRNAs were differentially expressed in NSCLC tissues (Fold change cut-off: 5.0, *P* < 0.05), including 304 upregulated and 267 downregulated lncRNAs. Table 1 showed 14 lncRNAs randomly selected among the differential lncRNAs (Log2 fold change, *P* < 0.05).

**Table 1:** Part of lncRNAs detected using microarray in three NSCLC patients

Upregulated in cancer		Downregulated in cancer	
lncRNAs	Log_2_ fold change (T/N)	lncRNAs	Log_2_ fold change (T/N)
LOC145757	4.1850148	ANKRD20A5P	−2.416844
DLX6-AS1	3.893559	C22orf34	−2.4658422
LOC284801	3.6185247	MAGI2-AS3	−2.565988
KIAA1908	3.4127824	LOC100505495	−3.384433
XLOC_008466	3.2270826	MGC27382	−4.104419
PRSS30P	3.149014	LOC400550	−4.5237017
LINC00665	2.945509	XLOC_001412	−4.6995843
RGMB-AS1	2.6495147	XLOC_l2_006399	−4.907096
HOTAIR	2.581743	RP11-165H20.1	−5.3639335

T: NSCLC tissues; N: adjacent normal tissues

### Expression of lncRNA RGMB-AS1 and RGMB mRNA in NSCLC tissues

**Fig. 1:**
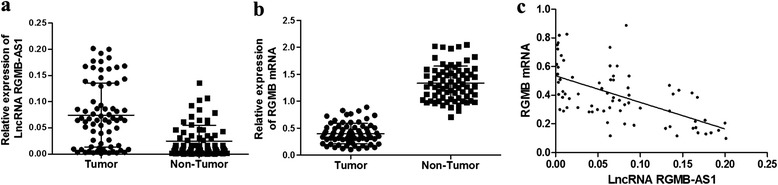
Expressions of lncRNA RGMB-AS1 and RGMB mRNA in NSCLC tissues and adjacent normal tissues. **a** Relative expression of lncRNA RGMB-AS1 in paired NSCLC tissues (tumor group) and adjacent normal tissues(non-tumor group). There is a statistically significant difference (*P* < 0.05). **b** RGMB mRNA expression in the tumor group was notably higher than in non-tumor group (*P* < 0.05). **c** The expressions of lncRNA RGMB-AS1 and RGMB mRNA were inversely correlated (R^2^ = 0.590, *P* < 0.05)

In 72 NSCLC tissues, we performed qRT-PCR to check lncRNA RGMB-AS1 and RGMB mRNA expression, and found that lncRNA RGMB-AS1 expression was significantly higher in NSCLC tissues than in adjacent normal tissues (*P* < 0.05, Fig. 1a), while RGMB mRNA showed an opposite trend (*P* < 0.05, Fig. 1b). These data indicated that the expression of lncRNA RGMB-AS1 and RGMB mRNA were inversely correlated (R^2^ = 0.590, *P* < 0.05, Fig. 1c).

### Association of lncRNA RGMB-AS1 and RGMB expression with clinicopathologic features of NSCLC patients

**Fig. 2:**
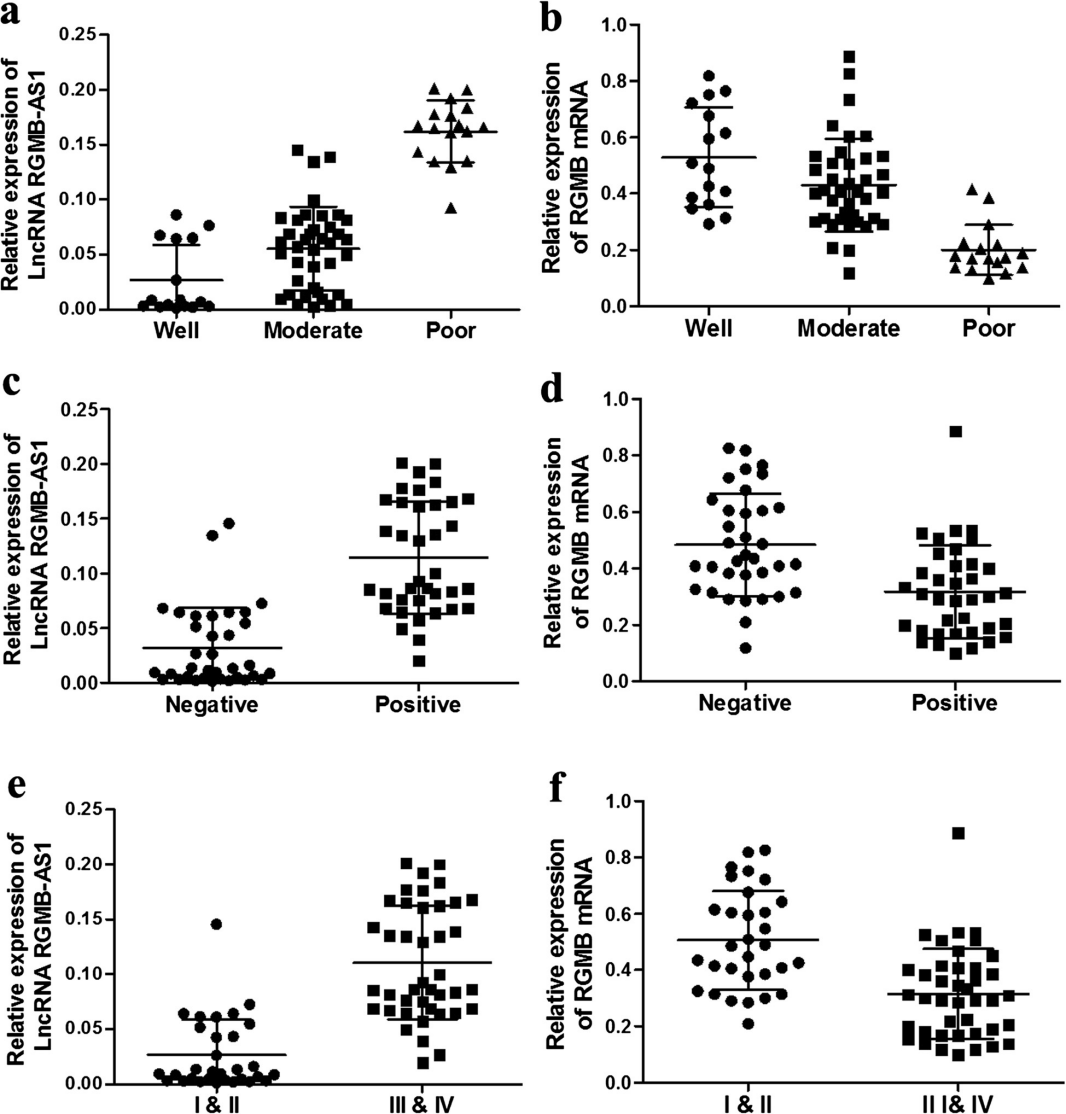
The correlation of lncRNA RGMB-AS1 and RGMB mRNA expression levels in NSCLC tissues. **a**, **b**: In poor differentiation tumor tissues, the expression levels of lncRNA RGMB-AS1 were significantly higher while RGMB mRNA were lower than in well and moderate differentiation tissues (*P* < 0.05). **c**, **d**: NSCLC tissues were divided into two groups of metastasis-positive and metastasis-negative. The high expression of lncRNA RGMB-AS1 and low expression of RGMB mRNA were notably associated with lymph node metastasis (*P* < 0.05). **e**, **f**: Significant differences were observed in lncRNA RGMB-AS1 and RGMB mRNA expression in different TNM stages (*P* < 0.05). *P* < 0.05 compared with the control group

Using the qRT–PCR analysis and with adjacent normal tissues as reference, the clinicopathologic features of the 72 NSCLC samples included in this study are presented in Table 2. We found that lncRNA RGMB-AS1 and RGMB expression levels in NSCLC tissues were associated with the occurrence of differentiation status, lymph node metastases and TNM stage (*P* < 0.05; Table 2, Fig. 2). No significant differences were observed between lncRNA RGMB-AS1, RGMB expression and either gender or age (*P* >0.05; Table 2).

**Table 2:** Association of lncRNA RGMB-AS1 and RGMB expression with clinicopathologic features of NSCLC patients

Clinicopathological factor	n	LncRNA RGMB-AS1 expression(2^–ΔCt^)		RGMB mRNA expression(2^–ΔCt^)	
		Median ± SD	*P*	Media ± SD	*P*
Gender					
Male	47	0.0734 ± 0.0641	0.847	0.4148 ± 0.2016	0.301
Female	25	0.0763 ± 0.0557		0.3656 ± 0.1684	
Age(years)					
≥60	28	0.0634 ± 0.0638	0.226	0.4320 ± 0.2111	0.227
<60	44	0.0814 ± 0.0587		0.3759 ± 0.1761	
Histology					
SCC	44	0.0718 ± 0.0584	0.662	0.4020 ± 0.1989	0.812
Adeno	28	00784 ± 0.0656		0.3909 ± 0.1813	
Differentiation					
Well	16	0.0269 ± 0.0323	0.007*	0.5292 ± 0.1771	0.032*
Moderate	39	0.0556 ± 0.0378		0.4299 *±* 0.1633	
Poor	17	0.1622 ± 0.0281		0.2000 ± 0.0883	
Lymphnode metastasis					
Positive	37	0.1146 ± 0.0514	0.000*	0.3169 ± 0.1640	0.000*
Negative	35	0.0319 ± 0.0367		0.4832 ± 0.1819	
TNM					
I + II	31	0.0265 ± 0.0326	0.000*	0.5068 ± 0.1751	0.007*
III + IV	41	0.1106 ± 0.0517		0.3152 ± 0.1598	

SCC: Squamous cell carcinoma; Adeno: Adenocarcinoma

*Indicated statistical significance (*P* < 0.05)

### siRNA of lncRNA RGMB-AS1 can promote the expression of RGMB in A549 and SPC-A-1 cells

**Fig. 3:**
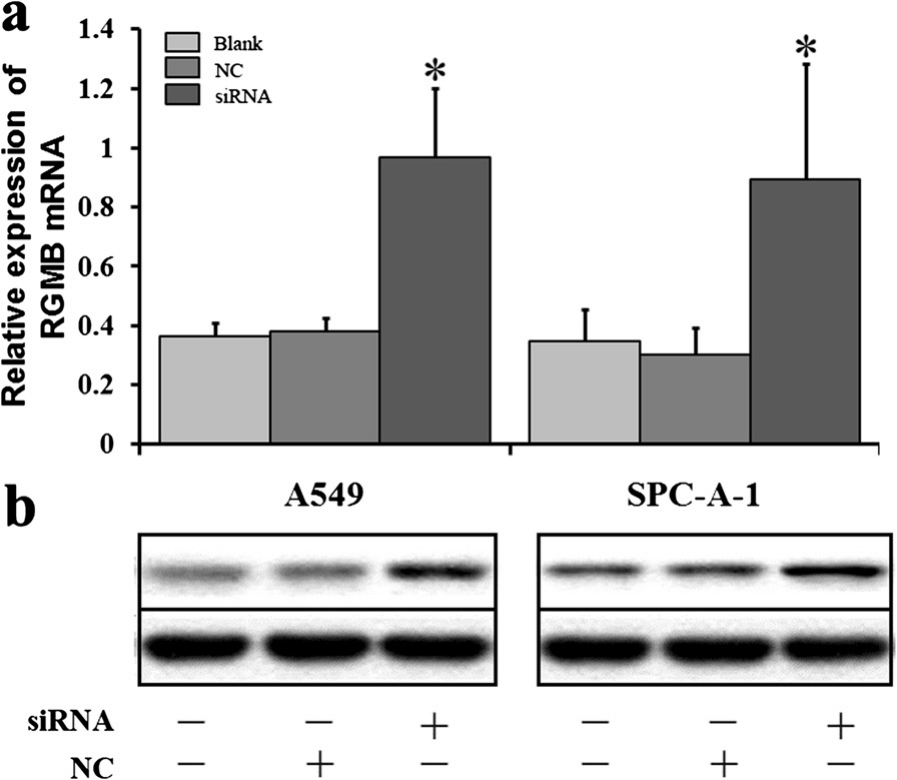
siRNA of lncRNA RGMB-AS1 promoted the expression of RGMB in A549 and SPC-A-1 cells. **a** Cells were transfected with siRNA of lncRNA RGMB-AS1 and NC. RGMB mRNA level was detected by qRT-PCR assay. RGMB mRNA expression was upregulated in A549 and SPC-A-1 cells after transfection with siRNA of lncRNA RGMB-AS1 and NC respectively (*P < 0.05). **b**, RGMB protein level was detected by Western blot assay. GAPDH protein was regarded as endogenous normalize. The expression level was also upregulated (*P < 0.05). siRNA: cells transfected with siRNA of lncRNA RGMB-AS1; NC: cells transfected with unrelated siRNA as negative control; Blank: non-transfected cells. *P < 0.05 compared with the control group

To further study that lncRNA RGMB-AS1 expression has an opposite correlation with RGMB expression, we studied whether knockdown of lncRNA RGMB-AS1 would have an effect on the expression of RGMB. Transfection with siRNA of lncRNA RGMB-AS1, subsequent qRT-PCR and western blot analysis indeed showed that RGMB mRNA (*P* < 0.05, Fig. 3a) and protein expression (*P* < 0.05, Fig. 3b) were upregulated in A549 and SPC-A-1 cells compared to the control groups.

## Discussion

The relationship between lncRNAs and tumors has currently become one of the focuses of cancer studies. In digestive system tumors, lncRNA HNF1A-AS1 has been shown to regulate proliferation and migration in oesophageal adenocarcinoma cells [21]. LncRNA HOTAIR was found overexpression in human hepatocellular carcinomas (HCC) [22]. The highly upregulated in liver cancer (HULC) gene expression is confined to colorectal carcinomas that metastasize to the liver [23]. In hematological system tumors, lncRNA NEAT1 expression has been revealed to impair myeloid differentiation in acute promyelocytic leukemia cells [24]. In urinary system tumors, there have been reports in recent years about lncRNA H19 associating with bladder cancer [25, 26]. In the male reproductive system, prostate cancer gene expression marker 1 (PCGEM1) has been demonstrated as a prostate tissue-specific, and prostate cancer-associated noncoding RNA (ncRNA) gene [27]. In respiratory system tumors, MALAT1 was originally identified as a prognostic marker for metastasis and patient survival in NSCLC, specifically in early stages of lung adenocarcinoma [28]. In this study, we have identified lncRNA RGMB-AS1 is aberrantly expressed in human NSCLC tissues compared to paired adjacent normal tissues. We also found that altered lncRNA RGMB-AS1 expression levels are associated with the occurrence of lymph node metastases, and the differentiation status and TNM stage in NSCLC patients, which may be a hint for NSCLC occurrence and be a potential biomarker for the diagnosis of early NSCLC.

Simultaneously, we also identified RGMB is downregulated in human NSCLC tissues via qRT-PCR analysis. Statistical analysis showed RGMB expression was also related to the occurrence of lymph node metastases, and the differentiation status and TNM stage in NSCLC patients. RGMB, also known as DRAGON, is a member of the repulsive guidance molecules (RGMs) family which consists of RGMA, RGMB, and RGMC [29]. RGMs are a group of cysteine rich 33 kDa proteins, including an N-terminal signal peptide, proteolytic cleavage site, partial von Willebrand factor type D domain, and glycophosphatidylinositol (GPI) anchor [30, 31]. RGM proteins can function as bone morphogenetic protein (BMP) coreceptors [32–34], which are members of the transforming growth factor beta (TGF-β) family of ligands and play a role in many biological activities. Specifically, RGMB directly interacts with BMP receptors for BMP-2 and BMP-4 enhancing binding to their ligands [35]. RGMs work

as BMP co-receptors and by participating in BMP signalling pathway may also be involved in cancer development and progression. In prostate cancer, the knockdown of RGMB significantly enhanced the prostate cancer cell capacity, namely increased growth, adhesive, motility and mobility [36]. Knockdown of RGMB also was studied in breast cancer. The results showed the promotion of growth, survival, adhesion, and migration of breast cancer cells [37, 38].

The UCSC data-base results showed that lncRNA RGMB-AS1 was localized in human chromosome 5 between 98105322 and 98108829 base sites. To check the location of RGMB, UCSC data-base also showed that it was localized in human chromosome 5 between 98109606 and 98129556 base sites. The locations of both lead us to infer that lncRNA RGMB-AS1 and RGMB may have a certain expression control. Applying statistical analysis and performing RNA interference technique in A549 and SPC-A-1 cells, we revealed that lncRNA RGMB-AS1 expression was inversely associated with RGMB expression. The detailed molecular analysis of lncRNA RGMB-AS1 and RGMB still need to be further studied.

## Conclusions

In summary, we identified upregulation of lncRNA RGMB-AS1 and downregulation of RGMB in 72 NSCLC patients, and both were related to differentiation status, lymph node metastases and TNM stage. We also provided evidence demonstrating the probable association between lncRNA RGMB-AS1 and RGMB. Based on these findings, we propose that lncRNA RGMB-AS1 and RGMB may serve as a new direction in NSCLC research. But more elaborate studies will be necessary for further exploration of the potential role of lncRNA RGMB-AS1 and RGMB in development of NSCLC in the future.

## Abbreviations

NSCLC: Non-small-cell lung cancers
lncRNAs: Long non-coding RNAs
nt: Nucleotides
RGMB: Repulsive guidance molecule b
SCC: Squamous cell carcinoma
Adeno: Adenocarcinoma
HCC: Human hepatocellular carcinomas
HULC: The highly upregulated in liver cancer
PCGEM1: Prostate cancer gene expression marker 1
GPI: Glycophosphatidylinositol
BMP: Bone morphogenetic protein
TGF-β: Transforming growth factor beta
